# A prospective study of physician pre-hospital anaesthesia in trauma patients: the rate of oesophageal intubation, gross airway contamination and the value of the ‘quick look’ airway assessment

**DOI:** 10.1186/1757-7241-21-S1-S26

**Published:** 2013-05-28

**Authors:** DJ Lockey, P Avery, T Harris, GE Davies, HM Lossius

**Affiliations:** 1 London’s Air Ambulance, Department of Pre-hospital Care, Royal London Hospital, London E1 1BB, UK; 2 Department of Research and Development, The Norwegian Air Ambulance Foundation, Holterveien 24, PO Box 94, N-1441 Drøbak, Norway; 3 Field of Pre-hospital Critical Care, Network of Medical Sciences, University of Stavanger, Kjell Arholmsgate 41, NO-4036 Stavanger, Norway

## Background

On the controversial background of advanced pre-hospital airway management many European EMS systems provide physicians to perform intubation on scene. We prospectively examined the rate of misplaced tracheal tubes, the presence and nature of gross airway contamination and the value of ‘quick look’ airway assessment to identify patients with subsequent difficult laryngoscopy.

## Methods

Patients requiring pre-hospital intubation in a physician–led pre-hospital trauma service over 16 months were included. Intubation success rate, misplaced tracheal tube rate, Cormack and Lehane grade and the presence and nature of gross airway contamination were recorded at laryngoscopy. Tube placement was verified with carbon dioxide detection and chest x-ray. After visual assessment physicians stated whether laryngoscopy was expected to be a straightforward or ‘difficult’. The assessment was compared to subsequent laryngoscopy grade.

## Results

400 patients had attempted intubation. 42 were in cardiac arrest and intubated without drugs. Of 399 successfully intubated patients there were no oesophageal or misplaced tracheal tubes. Gross airway contamination was reported in 179 of 402 patients (45%), of which ¾ was from the upper airway. Unconscious patients had higher contamination rates (55%) than conscious patients (28.8%) (p<0.0001). As a test of difficult intubation, the ‘quick look’ generated sensitivity 0.597 and specificity 0.763 (PPV and NPV were 0.336 and 0.904 respectively, with a likelihood ratio of 2.52).

## Conclusion

This study suggests that when physicians perform pre-hospital anaesthesia in a reproducible manner with carbon dioxide detection devices they have high intubation success rates and reduce or eliminate undetected misplaced tracheal tubes. We found high rates of airway contamination, mostly blood from the upper airway. The ‘quick look’ airway assessment had some utility but is unreliable in isolation.

